# The Indole–AhR–Treg axis as a microbiome-encoded immune checkpoint in type 1 diabetes

**DOI:** 10.3389/fimmu.2026.1949305

**Published:** 2026-10-30

**Authors:** Kaylee Delgado, Alexia Espitia, Kate PopeJoy, Lila Mace, Michelle Lew, Benny Shone Song, Rakesh Kumar, Jianxun Song

**Affiliations:** 1 Department of Microbial Pathogenesis and Immunology, Texas A&M University Health Science Center, Bryan, TX, United States; 2 Department of Biochemistry and Molecular Biology, School of Medicine, Louisiana State University Health Sciences Center, Shreveport, LA, United States; 3 Feist-Weiller Cancer Center, Louisiana State University Health Sciences Center, Shreveport, LA, United States

**Keywords:** aryl hydrocarbon receptor, dysbiosis, engineered bacterial therapeutics, gut microbiome, immune tolerance, indole metabolites, NOD mouse, regulatory T cells

## Abstract

Type 1 diabetes (T1D) is an autoimmune disease driven by the progressive destruction of pancreatic β-cells following failure of peripheral immune tolerance. Increasing evidence identifies the gut microbiome as a critical upstream regulator of this process through its control of microbiota-derived immunoregulatory metabolites. Among these, tryptophan-derived indole metabolites have emerged as central modulators of the aryl hydrocarbon receptor (AhR)–regulatory T cell (T_reg_) axis, a key checkpoint governing mucosal and systemic immune homeostasis. In individuals at risk for T1D, gut dysbiosis has been associated with altered commensal composition and tryptophan metabolism that may reduce the availability of selected AhR-active indole metabolites, including indole-3-acetic acid (IAA), indole-3-carboxaldehyde (ICA), and indole-3-propionic acid (IPA). These metabolites regulate T_reg_ differentiation, macrophage polarization, epithelial barrier integrity, and effector T cell activation through context-dependent AhR- and PPAR-mediated signaling pathways. Disruption of this axis promotes inflammatory Th1/Th17 responses, intestinal barrier dysfunction, and progression of islet autoimmunity. A central concept emerging from recent studies is that the immunological outcome of AhR activation depends not only on ligand identity, but also on delivery compartment, concentration, cellular target, and inflammatory context — a framework we term the “Indole Paradox.” This paradigm helps reconcile why some AhR-targeting interventions exacerbate autoimmunity whereas others restore immune tolerance. In this review, we synthesize current mechanistic and translational evidence linking microbiome-derived indole signaling to T1D pathogenesis and evaluate emerging therapeutic strategies targeting this axis, including dietary modulation, postbiotic metabolites, pharmacological AhR agonists, fecal microbiota transplantation, and engineered bacterial therapeutics. We propose that context-directed modulation of the indole–AhR–T_reg_ axis represents a promising next-generation strategy for restoring immune tolerance and preventing autoimmune progression in T1D.

## Graphical Abstract

**Figure:**
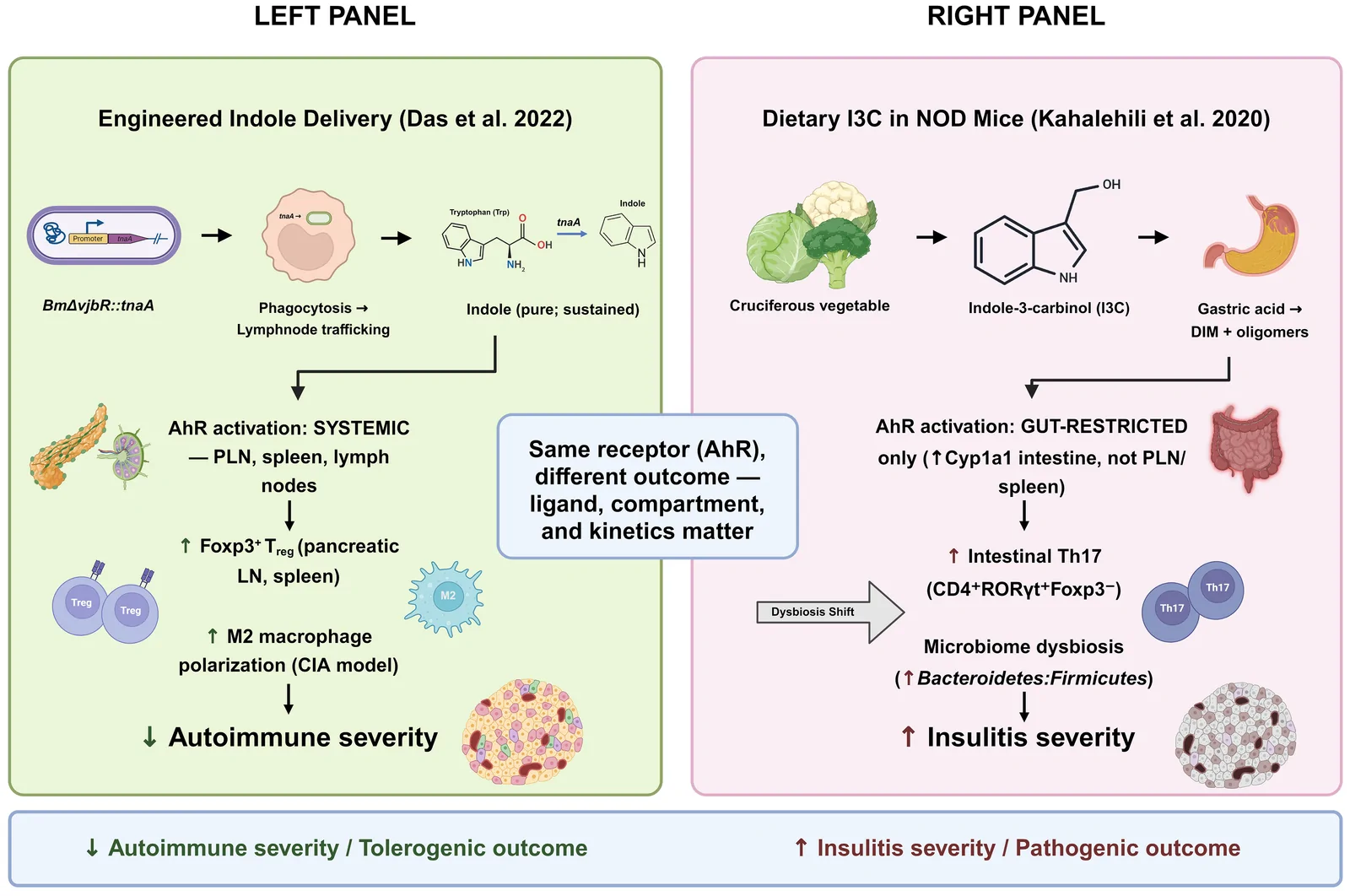
Microbiome-derived indole metabolites regulate immune tolerance through context-dependent activation of the aryl hydrocarbon receptor (AhR) and stabilization of Foxp3^+^ T_regs_. Under homeostatic conditions, indole-producing commensal bacteria promote epithelial barrier integrity, tolerogenic immune signaling, and suppression of pathogenic Th1/Th17 and autoreactive CD8^+^ T cell responses. In T1D, gut dysbiosis reduces indole production, disrupts AhR-mediated immune regulation, and promotes inflammatory amplification and β-cell destruction. The immunological consequences of AhR activation depend on ligand identity, tissue compartment, inflammatory context, and disease stage — a framework referred to as the “Indole Paradox.” Emerging therapeutic approaches aim to restore microbiome-mediated immune tolerance through dietary interventions, postbiotics, AhR agonists, fecal microbiota transplantation, and engineered bacterial therapeutics.

## 1 Introduction

Type 1 diabetes (T1D) is a chronic autoimmune disease characterized by progressive immune-mediated destruction of insulin-producing pancreatic β-cells, resulting in lifelong dependence on exogenous insulin therapy (1). Despite major advances in glucose monitoring and insulin replacement strategies, current treatments do not address the underlying failure of immune tolerance that drives disease progression (2–7). Moreover, the global incidence of T1D continues to rise, particularly among children and adolescents, at a rate that cannot be explained by genetic susceptibility alone. These observations strongly implicate environmental and host–microbe interactions as central contributors to autoimmune initiation and progression (8–10).

Among environmental regulators of immune homeostasis, the gut microbiome has emerged as a critical determinant of autoimmune susceptibility (3, 4). The intestinal microbiota continuously shapes mucosal and systemic immunity through microbial-associated molecular patterns, metabolic signaling, and modulation of epithelial barrier integrity. Longitudinal human cohort studies and preclinical models consistently demonstrate that gut dysbiosis precedes islet autoantibody development and overt diabetes onset. Individuals at risk for T1D frequently exhibit reduced microbial diversity, depletion of immunoregulatory commensal populations, impaired intestinal barrier function, and altered microbial metabolite production. These findings support the concept that disruption of microbiome-mediated immune regulation may contribute to the breakdown of peripheral tolerance in T1D (3, 4, 11).

Recent attention has increasingly focused on microbiota-derived tryptophan metabolites as key mediators linking gut microbial ecology to host immune regulation (12, 13). Dietary tryptophan can be metabolized by commensal bacteria into a diverse array of indole derivatives, including indole, indole-3-acetic acid (IAA), indole-3-propionic acid (IPA), indole-3-carboxaldehyde (ICA), indole-3-lactic acid (ILA), and related compounds. These metabolites regulate epithelial barrier integrity, innate immune activation, antigen-presenting cell function, macrophage polarization, and T cell differentiation through activation of the aryl hydrocarbon receptor (AhR), pregnane X receptor (PXR), and additional metabolic signaling pathways. Among these mechanisms, the AhR–T_reg_ axis has emerged as a particularly important immune checkpoint governing the balance between immune tolerance and inflammatory activation (6, 14).

The therapeutic potential of this axis has been complicated by a critical pharmacological paradox. The Indole Paradox — the observation that exogenous indole delivery can paradoxically drive Th17 expansion and worsen autoimmunity under specific conditions — has exposed fundamental limitations of early dietary and supplemental strategies and has motivated development of increasingly sophisticated delivery platforms aiming to achieve compartment-specific, concentration-controlled AhR activation (15–17). The present review provides a comprehensive synthesis of the mechanistic evidence supporting the indole-AhR-T_reg_ axis in T1D, evaluates the full spectrum of emerging therapeutic strategies targeting this axis, and identifies the experimental and translational requirements for their advancement.

## 2 Immunopathology of type 1 diabetes: the NOD mouse model and the T_reg_/T_eff_ axis

### 2.1 The NOD mouse as a translational model

The non-obese diabetic (NOD) mouse remains the most extensively characterized spontaneous model of autoimmune T1D and the standard pre-clinical platform for evaluation of immune-targeted therapies (2, 18). NOD mice develop progressive islet-specific autoimmunity beginning at 3–5 weeks of age, when macrophages and dendritic cells (DCs) infiltrate the peri-islet space (peri-insulitis); this transitions to destructive intra-islet insulitis between 8–12 weeks, with overt hyperglycemia occurring in 60-80% of female mice by 30 weeks (2, 18). Female NOD mice develop diabetes more frequently than males, a sex bias linked in part to reciprocal interactions between androgens and the gut microbiota (19). In NOD mice, transfer of an adult male microbiota to immature females increased testosterone, reduced islet inflammation and autoantibody production, and conferred androgen receptor-dependent protection from diabetes (19). In contrast, human T1D occurs in both sexes, and epidemiological studies commonly report either an approximately balanced distribution or a modest male predominance, particularly after puberty (20). Thus, sex-specific mechanisms identified in NOD mice should be considered a model-dependent variable rather than assumed to translate directly to patients. The potential implications of sex for AhR-targeted interventions are discussed in Section 6.2. The four-stage insulitis progression and the T_reg_/T_eff_ crossover at approximately week eight that define the closing therapeutic window are illustrated in **Figure 1** (see **Supplementary Table S3** for the stage-anchored derivation and data sources). The model’s utility derives from its deep genetic and immunological homology with human T1D: both share class II MHC susceptibility alleles (HLA-DR3/DR4-DQ2/DQ8 in humans; the I-Ag7 haplotype in NOD mice), autoreactive T cell responses targeting overlapping β-cell antigens including insulin, GAD65, and ZnT8, and a quantitatively normal but functionally impaired Foxp3^+^ T_reg_ compartment progressively outcompeted by expanding autoreactive effector populations (18, 21). A recent single-cell multi-omics study of human pancreatic lymph node tissue (pLN) from autoantibody-positive individuals confirmed that tissue-restricted T_reg_ deficits observed in NOD pLNs are present in human pre-T1D pLN tissue, validating the translational relevance of the model for the lymphoid immunopathology preceding clinical disease (22).

**Figure 1:**
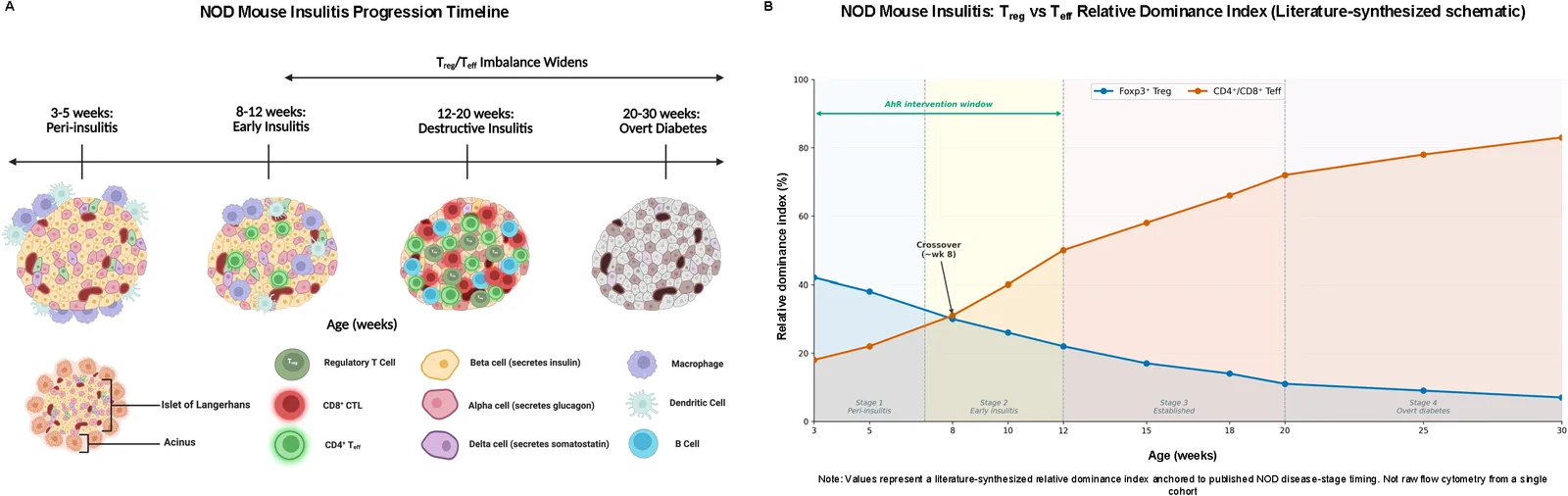
Progressive T_reg_ destabilization defines the therapeutic intervention window in T1D. **(A)** Schematic timeline of insulitis progression in the non-obese diabetic (NOD) mouse model, illustrating the transition from peri-insulitis to overt diabetes. Early disease stages are characterized by partial preservation of Foxp3^+^ regulatory T cell (T_reg_) function, whereas later stages exhibit progressive dominance of autoreactive effector T cells (T_effs_), inflammatory cytokine amplification, and β-cell destruction. **(B)** Literature-synthesized relative dominance model illustrating the progressive shift from T_reg_-mediated immune regulation to pathogenic T_eff_ predominance during T1D progression. The approximate T_reg_/T_eff_ crossover during early insulitis highlights a critical therapeutic window during which microbiome-derived immunoregulatory interventions may remain effective. Values represent conceptual evidence-weighted synthesis derived from published NOD mouse studies and are not raw flow cytometric measurements from a single experimental cohort.

### 2.2 Antigen priming and cDC1-dependent CD8^+^ T cell activation

The initiation of T1D follows a sequential process involving islet-antigen capture, antigen presentation in the pLN, and activation of autoreactive T cells. In BDC2.5-transgenic NOD mice, islet-reactive CD4^+^ T-cell proliferation is detectable in the pLN 10–14 days before histological insulitis becomes visible, indicating that antigen-specific T-cell activation precedes immune infiltration (23). Complementary experiments using 8.3 TCR-transgenic CD8^+^ T cells demonstrated that cross-presentation of the beta-cell antigen IGRP by Batf3^+^ cDC1s requires WDFY4. In WDFY4-deficient NOD mice, autoreactive CD8^+^ T-cell priming was abolished, whereas BDC2.5 CD4^+^ T-cell priming remained intact and autoimmunity was largely restricted to non-destructive peri-insulitis (24). cDC1-dependent CD8^+^ T-cell priming is a key immunological event required for the development of progressive, destructive insulitis. AhR activation can promote tolerogenic programs in dendritic cells characterized by increased IDO1, IL-10, and retinoic-acid-generating activity and reduced inflammatory cytokine production (25, 26). These effects may indirectly favor T_reg_ induction and restrain effector T-cell priming. However, whether AhR-targeted interventions directly alter cDC1-mediated cross-presentation in the pLN has not been established in T1D and remains an important question for future investigation.

### 2.3 CD4^+^ and CD8^+^ effector T cells as executors of β-cell destruction

Once primed in the pLN, autoreactive T cells recirculate to the pancreas and mediate β-cell destruction through mechanistically distinct pathways. CD8^+^ cytotoxic T lymphocytes (CTLs) destroy β-cells through perforin-granzyme B-mediated cytolysis and Fas-FasL interactions (1, 24, 27). In the CD4^+^ T cell compartment, Th1 cells secreting IFN-γ directly potentiate CD8^+^-mediated cytotoxicity by upregulating β-cell MHC class I expression and inducing CXCL10 production (1). Th17 cells amplify rather than initiate insulitis: genetic deletion of IL-17 reduces susceptibility to autoimmune diabetes in NOD mice, attenuating antigen presentation and pathogenic T cell activation, though the extent of protection is incomplete and dependent on the degree of IL-17 ablation (28). These findings position the Th17 compartment as an inflammatory amplifier rather than an indispensable initiator of insulitis (28). The reciprocal relationship between Th17 expansion and T_reg_ functional competence — both modulated in part by AhR signaling — positions this receptor as a molecular pivot point within the insulitic lesion. AhR provides a context-dependent connection between effector and regulatory T-cell responses. Depending on ligand identity, cellular target, and cytokine environment, AhR activation can support either Foxp3^+^ and IL-10-producing regulatory programs or RORγt- and IL-17-associated Th17 differentiation (5, 29). AhR should therefore be viewed as a context-sensitive modulator of the T_reg_–Th17 balance rather than as a uniformly tolerogenic pathway.

### 2.4 The T_reg_/T_eff_ imbalance: functional insufficiency rather than numerical absence

The T_reg_/T_eff_ imbalance driving T1D reflects not a numerical deficit of Foxp3^+^ cells but a progressive failure of T_reg_ suppressive capacity coinciding with the emergence of T_eff_ resistance to regulation (30, 31). Three complementary mechanisms account for this failure. First, NOD mice carry a hypomorphic allele at the Il2 locus that reduces IL-2 production by approximately 50%, resulting in a T_reg_ pool enriched for the non-suppressive CD62L^-^ subset (21). Second, intra-islet T_regs_ exhibit reduced expression of LAG-3 — an inhibitory co-receptor that regulates T_reg_ metabolic fitness by controlling PI3K-dependent glycolytic and oxidative phosphorylation programs (32). Third, NOD conventional T cells (T_convs_) are intrinsically resistant to T_reg_-mediated suppression, proliferating approximately two-fold faster and requiring higher T_reg_: T_eff_ ratios to achieve equivalent suppression (21). Grebinoski et al. demonstrated that T_reg_ insufficiency in autoimmune diabetes is associated with selective loss of Neuropilin-1 on intra-islet T_regs_, supporting a role for this pathway in determining the progression or arrest of islet autoimmunity and strengthening the mechanistic rationale for T_reg_-targeted intervention (30). Human pancreatic lymph node tissue from T1D patients recapitulates these findings: direct examination of pLN biopsy specimens revealed marked Th17 cell expansion and quantitative functional defects in Foxp3^+^ T_regs_ relative to non-diabetic controls, confirming that the T_reg_/T_eff_ imbalance is not a rodent-specific artifact but a core feature of human T1D immunopathology (31). The literature-synthesized relative dominance index of T_reg_ and T_eff_ populations across disease stages depicted in **Figure 1B** is constructed from the stage-anchored data in **Supplementary Table S3**; values represent comparative evidence displays, not raw cytometric measurements from a single experimental cohort (2, 18, 21, 24, 28, 30).

### 2.5 Accessory cellular mechanisms: integration with the AhR-T_reg_ axis

The T_reg_ insufficiency described above operates within a broader multicellular environment that amplifies and sustains islet autoimmunity. B cells infiltrate the peri-islet compartment early and facilitate BCR-mediated antigen capture and MHC class II-dependent T-cell activation. The preservation of C-peptide following rituximab-mediated B-cell depletion supports the functional importance of this amplification pathway (33–35). Innate antigen-presenting cells within inflamed tissues produce IL-1β, TNF-α, IL-6, and type I interferons, creating an environment that opposes T_reg_ induction and favors effector-cell activation (36, 37). iNKT-cell deficiency or hypo responsiveness provides an additional regulatory defect in NOD mice (38–40), whereas cytokine-driven beta-cell stress generates modified insulin, GAD65, and IAPP epitopes that broaden the autoreactive T-cell repertoire (41, 42). These populations connect to the AhR–T_reg_ axis through both direct and indirect pathways. AhR activation in dendritic cells can promote IDO1-, IL-10-, and retinoic-acid-associated tolerogenic programs, thereby creating conditions favorable for T_reg_ induction (25, 26). In macrophages, AhR signaling can reduce inflammatory NF-kB activity and promote regulatory polarization (36, 43, 44). AhR-dependent reinforcement of epithelial barrier integrity may additionally reduce microbial-product-driven activation of innate cells, B cells, and effector T cells (45, 46). However, direct AhR-dependent regulation of B cells, iNKT cells, and stressed beta cells has not been established as a central mechanism in T1D. These populations are therefore considered components of the inflammatory network in which the AhR–T_reg_ axis operates rather than established direct targets of that axis.

### 2.6 NOD-to-human translation: convergence and key limitations

The NOD model recapitulates several important components of human T1D, including autoreactive CD4^+^ and CD8^+^ T-cell responses, functional T_reg_ insufficiency, B-cell-mediated amplification of pathogenic immunity, and beta-cell stress-associated neoepitope formation (2, 18, 47). However, mechanisms demonstrated directly in NOD mice, including WDFY4-dependent cDC1 cross-presentation and the temporal sequence of CD4^+^ and CD8^+^ T-cell priming, have not been established with equivalent resolution in humans. The model also has important limitations: diabetes incidence is strongly influenced by housing conditions and microbiome composition; the marked female predominance does not reproduce the more balanced or modestly male-biased human epidemiology; and the initiating autoantigen hierarchy is more heterogeneous in human T1D (18). Human studies support the biological plausibility of individual components of the AhR–T_reg_ axis. *In vitro* AhR activation can promote IL-10-producing human Tr1-like cells and, in the presence of TGF-β, functional Foxp3^+^ induced T_regs_ (47). Alterations in gut microbial composition and metabolic activity, including changes involving tryptophan-related pathways, have been reported in individuals with T1D (48, 49). Tillett et al. further showed that short-chain fatty acid biotherapy altered microbiome associated metabolic and AhR-ligand activity, while microbiota from responder participants delayed diabetes following transfer into germ free NOD mice (50). Nevertheless, direct evidence connecting microbiota-derived indole exposure, cell-specific AhR activation, and T_reg_ function in patients with T1D remains limited. The complete indole–AhR–T_reg_ pathway should therefore be considered a translationally plausible model supported by convergent evidence rather than an established causal mechanism in human T1D.

## 3 The tryptophan-indole-AhR axis: microbiota-encoded immune tolerance

### 3.1 Tryptophan as a metabolic nexus of gut-immune crosstalk

Tryptophan (Trp) is an essential aromatic amino acid that, beyond its primary role in protein synthesis, functions as a substrate for three metabolically and immunologically distinct pathways: the kynurenine pathway, the indole pathway, and the serotonin (5-HT) pathway (51, 52). Quantitatively, approximately 95% of Trp catabolism proceeds through the kynurenine pathway, with 1-2% directed to serotonin synthesis and 4-6% entering the microbially driven indole pathway (51). Despite this quantitative disparity, the indole pathway exerts immunological effects disproportionate to the substrate quantities it processes, primarily through the activation of AhR in immune and epithelial cells (13, 53). The three-branch tryptophan catabolism architecture and the selective collapse of the indole branch in T1D-associated dysbiosis are illustrated in **Figure 2A** (see **Supplementary Note S1** for construction methodology). The directionally normalized abundance index comparing healthy versus T1D/pre-T1D microbiome composition in **Figures 2B, C** is synthesized from TEDDY and INNODIA cohort data; values represent comparative evidence displays, not raw 16S read counts from a single dataset (see **Supplementary Table S2** for full taxon-level evidence summary) (3, 11).

**Figure 2:**
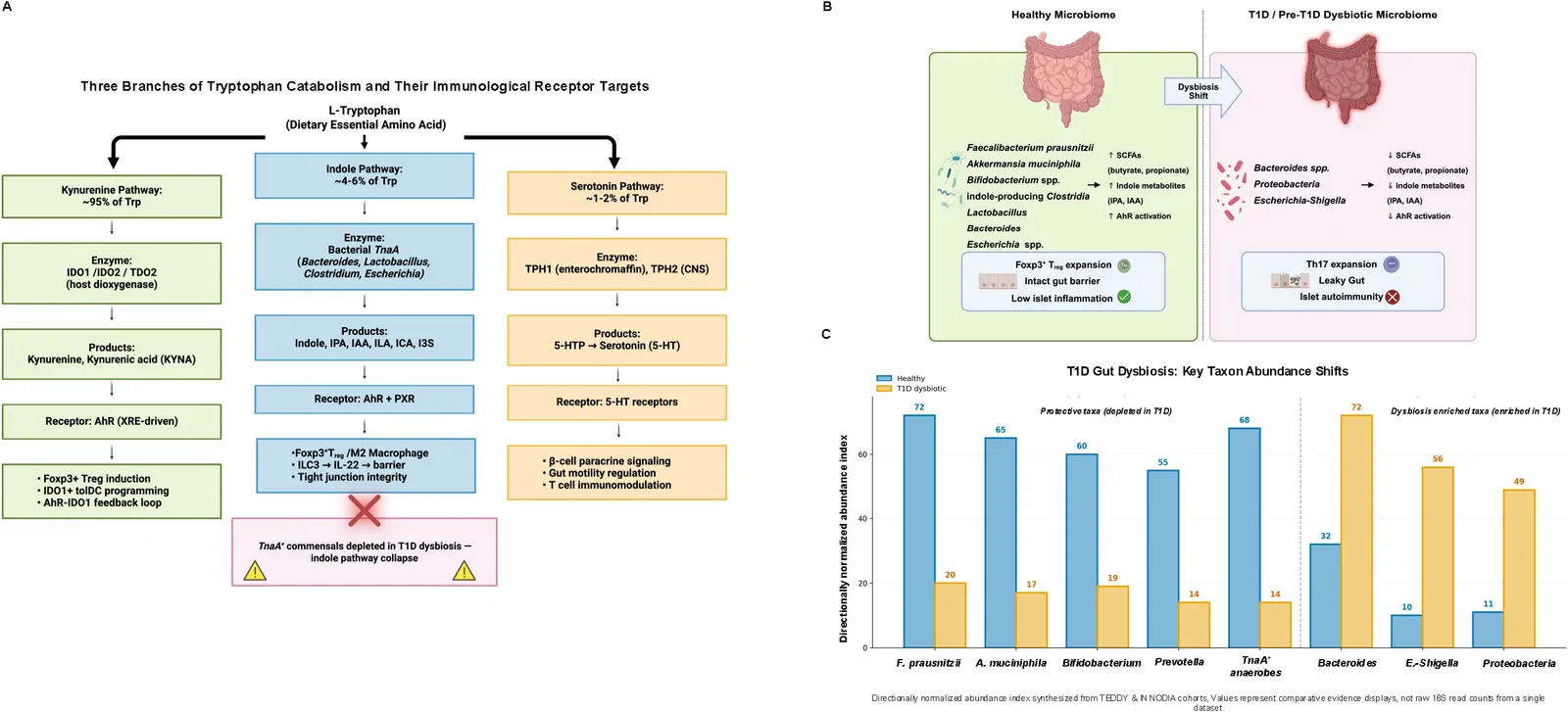
Microbiome-derived indole signaling regulates epithelial and immune homeostasis in T1D. **(A)** Commensal bacteria metabolize dietary tryptophan into a diverse array of indole derivatives, including indole, indole-3-acetic acid (IAA), indole-3-propionic acid (IPA), indole-3-carboxaldehyde (ICA), and related metabolites through microbial tryptophanase-dependent metabolic pathways. **(B)** Under homeostatic conditions, microbiota-derived indole metabolites activate AhR^-^, PXR^-^, and associated signaling pathways to promote epithelial barrier integrity, antimicrobial peptide production, tolerogenic dendritic cell programming, anti-inflammatory macrophage polarization, and Foxp3^+^ regulatory T cell (T_reg_) stability. **(C)** Dysbiosis-associated depletion of indole-producing commensal bacteria disrupts epithelial barrier function and reduces tolerogenic metabolite signaling, resulting in enhanced inflammatory cytokine production, pathogenic Th1/Th17 activation, autoreactive CD8^+^ T cell expansion, and autoimmune amplification during T1D progression.

### 3.2 The kynurenine pathway: tolerogenic enzyme machinery and inflammatory limits

The kynurenine pathway is initiated by three rate-limiting dioxygenases — IDO1, IDO2, and tryptophan 2,3-dioxygenase (TDO2) — each with functionally non-redundant roles (51). IDO1, predominantly expressed in DCs and macrophages, is driven by IFN-γ and TNF-α as part of an inflammation-sensing feedback mechanism. Kynurenine and kynurenic acid (KYNA) function as endogenous AhR ligands, triggering conformational change, nuclear translocation, Aryl Hydrocarbon Receptor Nuclear Translocator (ARNT) heterodimerization, and transcription of AhR response elements (XREs) (54). The net immunological output of IDO1-driven kynurenine production depends critically on the inflammatory state of the AhR-expressing cell: in homeostatic conditions, kynurenine-AhR reliably promotes tolerance, whereas in established inflammatory niches IDO1 overactivation can sustain a chronically immunosuppressive environment that fails to eliminate the autoimmune effector pool (25).

### 3.3 The serotonin pathway: bidirectional immune modulation

The serotonin pathway converts Trp to 5-HT via tryptophan hydroxylase (TPH1 in enterochromaffin cells; TPH2 in the central nervous system) (52). Approximately 95% of systemic 5-HT is produced in the gut under the influence of microbiota composition and SCFA availability (52). In T1D, β-cells express TPH1 and produce 5-HT as a paracrine signal regulating insulin secretion, such that autoimmune destruction disrupts a local serotonergic regulatory circuit (55, 56).

### 3.4 The indole pathway: microbiota-driven AhR activation and immune tolerance

The microbially driven indole pathway is initiated when commensal bacteria expressing the *tnaA* gene cleave dietary Trp into indole, pyruvate, and ammonia via a pyridoxal phosphate-dependent β-elimination reaction (12). The *tnaA* gene product is widely distributed across anaerobic commensals including *Bacteroides*, *Lactobacillus*, *Clostridium*, and *Escherichia species* (12). The principal indole derivatives of immunological importance are IPA, IAA, ILA, ICA, and indole-3-sulfate (I3S) — each engaging AhR, the pregnane X receptor (PXR), or both, with distinct potencies and downstream outcomes (14).

IPA is a potent PXR agonist and a weak-to-moderate AhR ligand whose immunological effects in CD4^+^ T cells are concentration-context dependent. Li et al. demonstrated that IPA stimulates mitochondrial respiration in CD4^+^ T cells by increasing fatty-acid and amino-acid oxidation while limiting glycolytic capacity. These effects were mediated by PPARβ/δ and were independent of AhR signaling in this experimental context, resulting in inhibition of Th1 and Th17 differentiation without impairing T_reg_ activity (7). IPA also upregulates tight junction proteins (ZO-1, occludin, claudins) in intestinal epithelial cells independently of AhR, reducing microbial LPS translocation (14). IAA expands Foxp3^+^ T_regs_ and suppresses CIA severity via the canonical AhR-ARNT-XRE transcriptional pathway (57). ICA drives AhR nuclear translocation in T lymphocytes, upregulates Pik3ip1 (a negative regulator of the PI3K/Akt/mTOR pathway), and suppresses effector T cell activation without impairing T_reg_ induction (58).

### 3.5 AhR structure, canonical signaling, and non-genomic mechanisms

The AhR is a ligand-activated transcription factor of the bHLH/PAS family (17). In its inactive cytosolic state, AhR exists within a chaperone complex comprising HSP90, XAP2 (AIP), and p23. Upon ligand engagement, nuclear AhR dimerizes with ARNT and the resulting heterodimer drives XRE-dependent transcription of immunologically relevant genes including CYP1A1, IL-10, IL-22, and Foxp3 (17, 59). Beyond canonical XRE-driven transcription, AhR participates in non-genomic signaling through direct interactions with NF-κB: the RelA (p65) subunit physically associates with activated AhR, and in myeloid cells this interaction targets RelA for ubiquitin-mediated proteasomal degradation, dampening NF-κB-dependent transcription of IL-1β, IL-6, and TNF-α and shifting macrophages from an M1-like toward an M2-like regulatory phenotype (36, 60).

### 3.6 AhR in adaptive immune cells: T cell fate decisions and the cytokine context rule

In CD4^+^ T cells, AhR activation modulates transcriptional programs governing effector versus regulatory fate decisions, with outcomes determined by ligand identity, cytokine milieu, and cellular activation state (60). In a tolerogenic microenvironment enriched with TGF-β and IL-2 — characteristic of the mucosal lamina propria and peri-pancreatic draining lymph nodes in non-inflammatory conditions — AhR activation by indole derivatives promotes Foxp3 expression and IL-10 secretion, consolidating T_reg_ commitment (5). De Juan et al. demonstrated in tumor-bearing mice that physiological AhR activation by food-derived ligands supported the efficacy of anti-PD-1 immunotherapy through a direct effect on CD8^+^ T cells, including progenitor-exhausted and effector CD8^+^ T-cell populations (61). This study did not demonstrate a direct T_reg_-mediated mechanism and is cited here as evidence that the immunological outcome of AhR signaling depends on the responding cell population and disease context.

In contrast, when AhR is activated in CD4^+^ T cells simultaneously stimulated by IL-6 and IL-1β — the cytokine environment characteristic of the insulitic islet — the balance shifts toward Th17 commitment, as AhR-driven STAT3-dependent transcription amplifies RORγt expression and IL-17 production (29). This bifurcation explains why the same AhR ligand can promote either tolerance or inflammation depending on whether it is encountered by a naive T cell in a homeostatic environment or by an activated T cell embedded in an inflammatory niche — the mechanistic basis of the Indole Paradox described in Section 3.8 (15).

### 3.7 AhR in dendritic cells, macrophages, and ILC3s: tolerogenic programming

In DCs, AhR activation induces a tolerogenic transcriptional program characterized by IDO1 upregulation, IL-12p70 suppression, IL-10 induction, and enhanced retinoic acid (RA) synthesis via Aldh1a2 (RALDH2) upregulation (26). The AhR-IDO1 positive feedback loop allows tolerogenic DCs to self-amplify their immunosuppressive programming (25). In macrophages, AhR activation shifts polarization from pro-inflammatory M1 to regulatory M2 phenotype via AhR-mediated upregulation of IRF4 (43, 44). In ILC3s, AhR activation by indole metabolites drives IL-22 production, reinforcing epithelial tight junction expression, inducing antimicrobial peptides (Reg3β, Reg3γ), and maintaining the physical separation of commensal bacteria from the epithelial surface (45, 46).

### 3.8 The indole paradox: contextual determinism of AhR activation

Evidence from autoimmune models illustrates how ligand identity, anatomical compartment, and disease context determine the consequences of indole-related signaling. Kahalehili et al. demonstrated that dietary I3C produced strong AhR activation in the small-intestinal mucosa of NOD mice, with little or no detectable activation in the pancreatic lymph nodes. The associated expansion of Th17 cells occurred in intestinal compartments, including the lamina propria, intraepithelial compartment, and Peyer’s patches, whereas the downstream disease outcome was increased pancreatic insulitis (15). In the collagen-induced arthritis model, Seymour et al. showed that microbiota-derived indole was generated in the intestinal or cecal compartment and promoted systemic inflammatory responses, including increased RORγt^+^ CD4^+^ T-cell and IL-17-associated responses, with downstream pathology occurring in the joints (16). These studies are therefore complementary rather than contradictory: both identify pro-inflammatory consequences, but they involve different indole-related exposures, immune compartments, and target-organ diseases. The relevant contrast is between these studies and reports in which selected indole derivatives or targeted AhR agonists promote T_reg_ or tolerogenic myeloid programs. Free indole or gut-restricted I3C exposure should therefore not be regarded as biologically equivalent to IPA, IAA, ICA, or cell-targeted AhR activation. Together, these findings support the central premise of the Indole Paradox: the outcome is determined by ligand identity, concentration, responding cell population, anatomical compartment, and inflammatory context. The contextual determinants governing AhR-driven T cell fate across five therapeutic modalities are summarized in the heatmap matrix in **Figure 3B** (see **Supplementary Table S4** for the full cell-level reference assignment logic).

**Figure 3:**
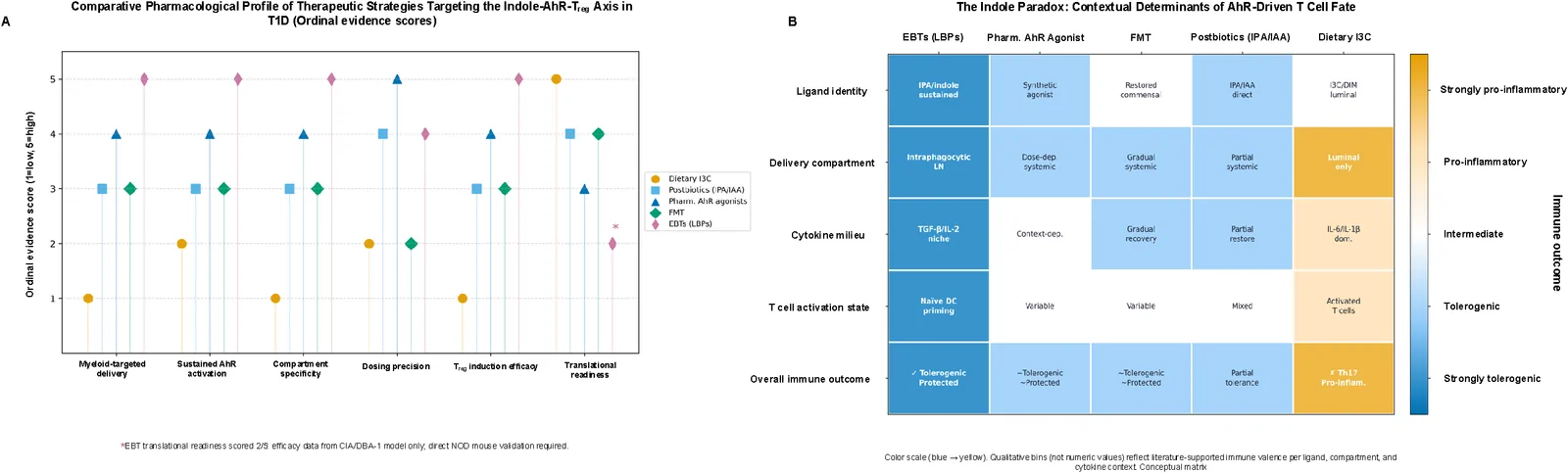
Comparative profiles and context-dependent immune effects of strategies targeting the indole–AhR–T_reg_ axis. **(A)** Literature-derived ordinal comparison of five therapeutic strategies—dietary indole-3-carbinol (I3C), purified postbiotic metabolites, pharmacological AhR agonists, fecal microbiota transplantation (FMT), and engineered bacterial therapeutics (EBTs)—across myeloid-targeted delivery, sustained AhR activation, compartment specificity, dosing precision, T_reg_-induction potential, and translational readiness. Scores are qualitative, evidence-weighted comparisons rather than measurements from a head-to-head experimental study. Scoring criteria and supporting references are provided in **Supplementary Table S1**. The EBT efficacy assessment is based on evidence obtained with *BmΔvjbR::tnaA* in the CIA/DBA/1 mouse model; direct efficacy in NOD mice remains to be established. **(B)** Conceptual matrix illustrating how ligand identity, delivery compartment, cytokine milieu, and responding-cell activation state may influence tolerogenic versus pro-inflammatory outcomes of AhR signaling across five intervention classes. Colors represent qualitative immune-valence categories rather than numerical effect sizes. The evidence assignments and supporting references are provided in **Supplementary Table S4**.

## 4 The T1D gut microbiome: dysbiosis as an upstream driver of islet autoimmunity

### 4.1 The compositional architecture of T1D-associated gut dysbiosis

The gut microbiome of individuals who develop T1D shows a convergent dysbiotic signature detectable months to years before clinical disease onset, consistent with a potential upstream contribution, although these temporal associations do not by themselves establish causality (11, 62). The landmark TEDDY study — analyzing 10,913 metagenomes from 783 infants across four countries — established that children progressing to T1D showed a functionally coherent deficit in fermentation capacity, characterized by reduced SCFA biosynthesis genes and butyrate-producing *Firmicutes*, with increasing *Bacteroides* dominance and *Escherichia/Shigella* abundance detectable even before the earliest islet autoantibodies appeared (3). The 2024 INNODIA longitudinal metagenomics study confirmed in 98 newly diagnosed T1D patients that gut microbiome diversity inversely correlated with C-peptide decline rate and that *Faecalibacterium prausnitzii* abundance predicted residual β-cell function at diagnosis, supporting a clinical association between microbiome features and metabolic disease progression (11). The directionally normalized abundance index for depleted and enriched taxa in T1D dysbiosis, illustrated in **Figure 2C**, is synthesized from TEDDY and INNODIA cohort data; full taxon-level evidence and references are provided in **Supplementary Table S2** (3, 11).

Yuan et al. demonstrated that fecal microbiota from T1D children transferred to antibiotic treated mice induces elevated fasting glucose and declined insulin sensitivity — supporting a functional contribution of the T1D associated microbiome in this recipient-mouse model (63). At the taxon level, the most consistently depleted genera are *Faecalibacterium, Akkermansia, Bifidobacterium, Prevotella*, and *Roseburia*, with reciprocal enrichment of *Bacteroides, Proteobacteria*, and *Escherichia-Shigella* (48).

### 4.2 *Faecalibacterium prausnitzii* and *Akkermansia muciniphila*

*Faecalibacterium prausnitzii* exerts anti-inflammatory effects through butyrate-mediated HDAC inhibition, secretion of microbial anti-inflammatory molecules (MAMs) suppressing NF-κB activation, and maintenance of intestinal tight junction protein expression that reduces LPS translocation (64). In T1D-specific contexts, *F. prausnitzii* depletion correlates with reduced peripheral T_reg_ frequencies, elevated IL-17A, and greater islet autoantibody burden across multiple pediatric cohort studies (64).

*Akkermansia muciniphila* reinforces barrier function by stimulating mucin production and maintaining goblet cell density, and its outer membrane protein Amuc_1100 activates TLR2-dependent intestinal immune training that reduces endotoxemia and systemic inflammatory tone (65). In T1D, *A. muciniphila* abundance is genetically correlated with autoantibody risk in GWAS-linked analyses, and its depletion from NOD mouse microbiomes at the pre-insulitic stage precedes a rise in circulating LPS-binding protein (65).

### 4.3 Critical windows for microbiome-mediated protection

Segmented filamentous bacteria (SFB) occupy a unique position in the NOD microbiome as potent inducers of intestinal Th17 cells whose presence can either protect against or accelerate T1D depending on the immunological context of colonization (62, 66). Fernandez Trigo et al. demonstrated that germ-free NOD mice colonized with SPF microbiota at birth were substantially protected from T1D, while colonization after weaning provided no protection — establishing that the immunoprotected window for microbiome-mediated T_reg_ programming is restricted to the early postnatal period (67).

### 4.4 Gut permeability, LPS translocation, and systemic immune activation

Depletion of barrier-reinforcing commensals is associated with structural breakdown of the intestinal epithelial tight junction network, resulting in increased paracellular permeability and translocation of LPS into portal and systemic circulation (68, 69). Zonulin — the principal physiological modulator of tight junction permeability — is measurably elevated in the serum of T1D patients and autoantibody-positive children, and elevated circulating zonulin precedes clinical diabetes onset in prospective cohort data (70).

### 4.5 SCFAs, the T_reg_/Th17 balance, and tryptophan metabolism: mechanistic integration

The immunoregulatory outputs of the gut microbiome converge on the T_reg_/Th17 balance through two intersecting metabolic networks: the SCFA axis and the tryptophan-indole axis. Butyrate-mediated HDAC inhibition increases acetylation of the Foxp3 promoter locus, stabilizing Foxp3 expression in differentiating naive T cells (71). Tillett et al. demonstrated that SCFA biotherapy delays diabetes in humanized gnotobiotic mice by remodeling gut mucosal proteome and metabolome, associated with expanded colonic and splenic T_regs_, reduced insulitis scores, and suppressed islet-reactive CD8^+^ T cell frequencies (50).

### 4.6 Human indole metabolomics: clinical evidence for the AhR pathway deficit

Human studies provide evidence that T1D is associated with altered microbiome composition, tryptophan metabolism, and selected indole-related metabolites, although the direction and causal significance of these changes remain incompletely resolved (48, 49). In the TEDDY cohort, functional microbial differences associated with progression to islet autoimmunity or T1D primarily included reduced fermentation and short-chain fatty acid biosynthesis capacity (3). In the INNODIA cohort, lower microbial diversity was associated with more rapid C-peptide decline, while Faecalibacterium prausnitzii abundance was inversely associated with HbA1c at diagnosis (11). Tillett et al. further showed that short-chain fatty acid biotherapy increased AhR-ligand activity in human fecal samples and that microbiota from responder participants delayed diabetes following transfer into germ-free NOD mice (50). Collectively, these findings support the biological and translational plausibility of microbiome–metabolite–AhR interactions in T1D. Nevertheless, direct evidence demonstrating that a microbiota-derived indole deficit causes cell-specific AhR dysfunction and impaired Treg function before human T1D onset remains limited. The complete indole–AhR–Treg pathway should therefore be considered a testable translational model supported by convergent evidence rather than an established causal mechanism in patients.

## 5 The therapeutic landscape targeting the indole-AhR-T_reg_ axis in T1D

### 5.1 Rationale and pharmacokinetic framework for targeted AhR activation

The mechanistic convergence of T1D immunopathology on the indole-AhR-T_reg_ axis presents a compelling therapeutic target. The Indole Paradox imposes a critical constraint: immune outcome is determined by the compartment of delivery, ligand identity, and cellular context — not merely by receptor engagement (15–17). Any viable therapeutic strategy must (1): deliver AhR-activating ligands to the myeloid cells governing T_reg_ induction rather than to the intestinal mucosa where Th17 induction predominates (2); achieve sustained, controlled ligand concentrations that drive tolerogenic rather than pro-inflammatory AhR programs; and (3) operate in the cellular context — predominantly naive DC priming and macrophage M2 polarization — that produces durable Foxp3^+^ T_reg_ induction. The comparative pharmacological profiles of five therapeutic strategies across six mechanistically relevant delivery dimensions are illustrated in **Figure 3A**; ordinal evidence scores are defined in **Supplementary Table S1**. The contextual determinism framework operationalizing the Indole Paradox across these modalities is illustrated in **Figure 3B**; full cell-level reference assignments are provided in **Supplementary Table S4**.

### 5.2 Dietary interventions and the indole paradox in practice

The earliest and most clinically accessible strategy for targeting the indole-AhR axis has been dietary modulation. Kahalehili et al. demonstrated that oral I3C supplementation in NOD mice produces strong luminal AhR activation confined to the small intestinal mucosa, significantly exacerbating insulitis and providing no detectable AhR activation in the pancreatic lymph nodes (15). These findings establish that oral indole precursor delivery lacks the pharmacokinetic properties necessary to achieve systemic tolerogenic AhR activation — the clearest demonstration of the Indole Paradox in a clinically relevant intervention model.

### 5.3 Purified postbiotic metabolite delivery: IPA, IAA, and ICA

Postbiotic therapy — the direct administration of purified microbial metabolites — circumvents the need for live microbial colonization and provides precise dosing control. Chen et al. demonstrated that exogenous IPA alleviates intestinal epithelial cell injury and corrects gut barrier defects via TLR4/NF-κB suppression, reducing paracellular permeability and systemic endotoxemia (72). Prasad et al. reported that IPA links gut dysfunction to downstream end-organ diabetic pathology — including retinopathy — and that restoring tryptophan metabolism via IPA serves as both a biomarker and a therapeutic target in diabetic complications (73). Li et al. showed tryptophan catabolites, including ICA, ameliorated immune-mediated hepatic inflammation through AhR agonism in T lymphocytes, upregulating Pik3ip1 and suppressing effector T cell activation without impairing T_reg_ induction (58). Despite these promising properties, postbiotic delivery faces critical pharmacokinetic limitations: short biological half-lives require continuous high-dose administration that risks the pro-Th17 outcomes associated with supraphysiological luminal concentrations (16).

### 5.4 Pharmacological AhR agonists: small-molecule approaches

Nanoparticles co-delivering the AhR ligand ITE and proinsulin suppressed autoimmune diabetes in 8-week-old NOD mice by inducing a tolerogenic dendritic-cell phenotype and increasing Foxp3^+^ T_reg_ differentiation (74). The recently characterized fluorescent AhR ligand AGT-5 demonstrated that prophylactic oral administration reduces hyperglycemia, decreases insulitis, and promotes tolerogenic DC (tolDC) and T_reg_ compartments in both pancreatic infiltrates and small intestinal lamina propria in a streptozotocin-induced murine T1D model (75). AGT-5-mediated effects were confirmed as AhR-dependent through Cyp1a1 upregulation and abrogation of tolDC effects by IDO1 inhibition, demonstrating that the AhR-IDO1-tolDC positive feedback loop can be pharmacologically activated in the T1D-relevant tissue microenvironment (75).

### 5.5 Fecal microbiota transplantation: restoring the ecological context

Unlike single-ligand interventions, fecal microbiota transplantation (FMT) seeks to modify the broader microbial ecosystem and its collective metabolic output, although its clinical effects cannot currently be attributed specifically to restoration of the indole–AhR–T_reg_ axis. In a randomized controlled trial of 20 adults with recent-onset T1D, de Groot et al. found that stimulated C-peptide was better preserved at 12 months after autologous FMT than after healthy-donor FMT. Baseline CD4^+^CXCR3^+^ T-cell counts and intestinal *Desulfovibrio piger* abundance were associated with preservation of residual β-cell function, suggesting that recipient-specific microbiome–immune interactions may influence treatment responses (76). In a separate randomized, double-blind, placebo-controlled pilot trial involving 20 adults with T1D and moderate-to-severe gastrointestinal symptoms, Høyer et al. found that encapsulated FMT was safe and improved gastrointestinal symptoms and quality of life compared with placebo (77). Høyer et al. subsequently reported that repeated, on-demand FMT provided sustained symptomatic benefit in individuals with T1D and severe diabetic gastroenteropathy (78). Collectively, these studies establish the clinical feasibility of FMT in selected T1D populations but do not demonstrate that its effects are mediated through restoration of the indole–AhR–T_reg_ axis; mechanistic biomarker studies are needed to test that connection.

### 5.6 Engineered bacterial therapeutics: precision platforms for intra-phagocytic immunomodulation

Engineered bacterial therapeutics (EBTs) are programmable live biotherapeutic products designed to synthesize or deliver immunomodulatory molecules at defined host–microbe or immune-cell interfaces (79, 80). Proof-of-concept for *L. lactis*-based EBTs in autoimmune diabetes was first established in NOD mice by Takiishi et al., who demonstrated reversal of established diabetes using antigen-secreting *Lactococcus lactis* combined with anti-CD3 treatment, producing sustained remission through restoration of antigen-specific tolerance in the pancreatic lymph node (81). Elias-Oliveira et al. demonstrated that *Lactococcus lactis* expressing heat shock protein 65 reduced T1D incidence from 80% to 50% in NOD mice (a 30 percentage-point absolute reduction), with preservation of islet architecture, and expansion of TLR2^+^cDC1^-^dependent T_reg_ populations, with upregulation of IDO1, Aldh1a2, Pdl1, and TGF-β in colonic tolerogenic DCs (81, 82). These molecular markers — IDO1, Aldh1a2 — are precisely those induced by indole-mediated AhR activation in DCs, revealing that multiple independent EBT platforms are converging on the AhR-IDO1-tolDC axis as the central molecular effector of microbiome-based immune tolerance induction (82).

*BmΔvjbR::tnaA*, an attenuated strain of *Brucella melitensis* metabolically reprogrammed via plasmid insertion of *Escherichia coli tnaA* to constitutively produce indole, was phagocytosed by circulating monocytes and provided sustained, intracellular indole delivery to lymph node-homing myeloid cells in a CIA model; a single administration polarized macrophages toward an anti-inflammatory IL-10-producing phenotype, promoted Foxp3^+^PD-1^+^ T_reg_ expansion, and dramatically reduced arthritis incidence and score — effects substantially amplified by co-administration with adoptive T_reg_ cell transfer (37). Critically, a single bolus dose of purified indole failed to replicate the EBT’s efficacy, establishing that sustained intracellular delivery — not peak concentration — is the operative therapeutic variable (37). The proposed cellular mechanism of action of *BmΔvjbR::tnaA* — including phagocytic trafficking to pLN, AhR activation in myeloid cells and T cells, and the convergent Foxp3^+^ T_reg_/M2 macrophage program — is illustrated in **Figure 4** (see **Supplementary Note S1** for the construction methodology and translational caveat).

**Figure 4:**
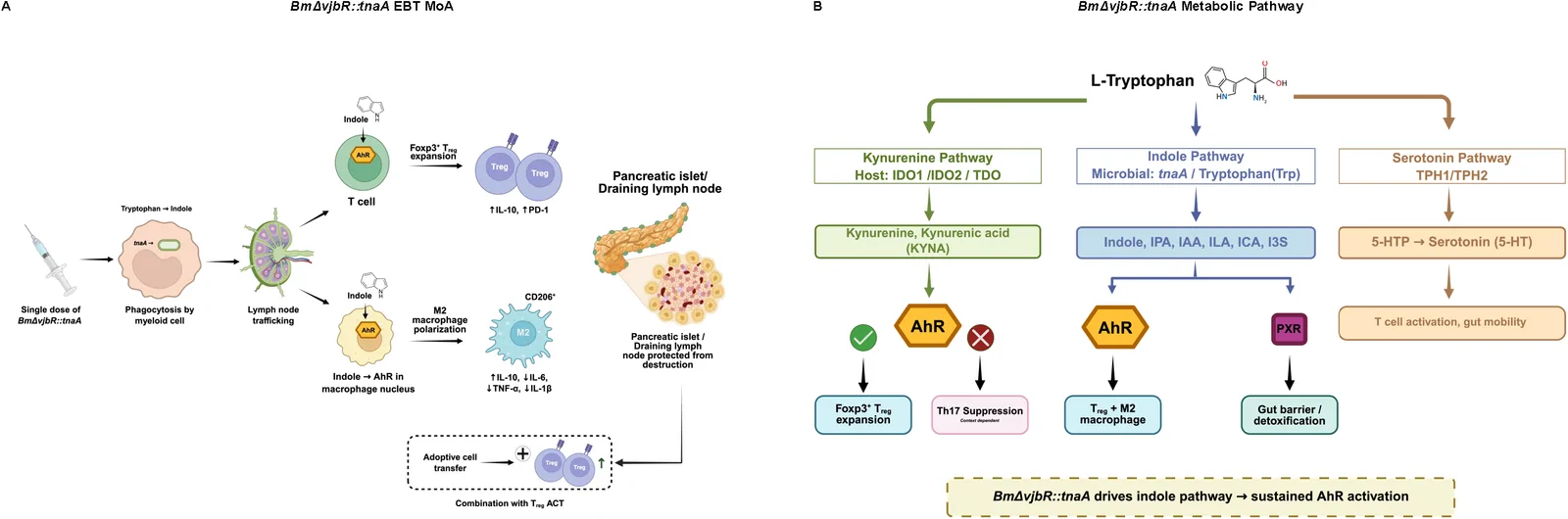
Proposed mechanism of context-directed indole delivery by the engineered bacterial therapeutic *BmΔvjbR::tnaA*. **(A)** Following administration, *BmΔvjbR::tnaA* is phagocytosed by myeloid cells, enabling sustained intracellular conversion of tryptophan to indole and trafficking to lymphoid tissues. Indole-mediated aryl hydrocarbon receptor (AhR) signaling is proposed to promote Foxp3^+^PD-1^+^ regulatory T-cell (T_reg_) expansion and an IL-10-associated regulatory/M2-like macrophage phenotype. In the collagen-induced arthritis (CIA) model, these immune responses were associated with reduced inflammatory disease and were enhanced by adoptive T_reg-_cell transfer. These mechanistic and efficacy findings were generated in male DBA/1 mice with CIA; application to pancreatic lymph nodes and autoimmune diabetes remains hypothetical and requires direct validation in non-obese diabetic (NOD) mice. **(B)** Tryptophan can be metabolized through the host kynurenine and serotonin pathways or the microbiota-dependent indole pathway. Expression of tnaA in *BmΔvjbR::tnaA* redirects tryptophan toward sustained intracellular indole production, enabling context-directed AhR signaling in phagocytic myeloid cells and associated regulatory immune programming. AhR outcomes remain dependent on ligand identity, cellular target, and inflammatory context.

All published mechanistic efficacy data for *BmΔvjbR::tnaA*, including T_reg_ expansion, macrophage M2 polarization, and arthritis score reduction, were generated exclusively in the CIA/DBA/1 male mouse model (37). Validation of the indole-producing EBT platform in the spontaneous NOD T1D model — and specifically confirmation that the tolerogenic myeloid-priming program is recapitulated in the pancreatic lymph node microenvironment — remains a critical and unmet experimental prerequisite before T1D-specific efficacy conclusions can be drawn.

### 5.7 Adoptive T_reg_ cell therapy and synergistic combinatorial approaches

Adoptive T_reg_ cell therapy (ACT) represents the most durable individual disease-modifying strategy currently in clinical development for T1D. Autologous polyclonal expanded T_reg_ cell therapy produced clinical benefit in a subset of T1D children in Phase I/II trials, with the most favorable outcomes in patients receiving two doses of T_regs_: 3 of 6 patients in remission versus 1 of 6 after one dose and 0 of 6 untreated controls, with C-peptide preservation and reduced exogenous insulin requirement maintained for up to 18–24 months in responders (83–85). Long-term follow-up established that remission duration is limited by the progressive inflammatory state and the IL-2 dependency of administered T_regs_, supporting the mechanistic case for pairing T_reg_ ACT with upstream AhR-mediated tolerogenic priming to provide a sustained permissive microenvironment (83–86). The synergistic efficacy of *BmΔvjbR::tnaA* when combined with ACT — substantially exceeding either strategy alone — identifies a mechanistically important combinatorial principle: microbiome-targeted AhR activation in myeloid cells may create the tolerogenic lymph node microenvironment that maximizes the inductive and stabilizing capacity for adoptively transferred or endogenously expanded T_regs_ (37, 83).

### 5.8 Comparative summary of therapeutic approaches

The five strategies targeting the indole-AhR-T_reg_ axis differ substantially in mechanism, delivery compartment, and translational maturity, as summarized in **Figure 3A** and **Table 1** (see **Supplementary Table S1** for the full ordinal evidence scoring rubric).

**Table 1:** Comparison of emerging therapeutic strategies targeting the indole–AhR–Treg axis.

Therapeutic strategy	Mechanism	AhR compartment	Key advantage	Key limitation
Dietary I3C	Luminal AhR activation via gastric hydrolysis	Intestinal only	Non-invasive; widely available	Indole Paradox: Th17 expansion; no pancreatic LN signal (15)
Postbiotics (IPA/IAA/ICA)	Direct PXR/AhR agonism	Systemic but rapid clearance	Bypasses colonization resistance	Short half-life; continuous high dose needed (72, 73)
Small-molecule AhR agonists (ITE, AGT-5)	Synthetic AhR agonism	Systemic; dose-dependent compartment selectivity	Precise dosing; clinical-stage feasibility	Concentration-dependent Th17 risk; limited spontaneous NOD data (75, 87)
FMT	Ecological microbiome restoration	Systemic via restored commensal community	Restores entire tryptophan metabolite ecosystem	Donor variability; autologous > allogeneic for C-peptide preservation (76)
EBTs (metabolite-producing LBPs)	Constitutive intracellular payload in myeloid cells	Intra-phagocytic; lymph node delivery	Sustained, myeloid-targeted; synergizes with T_reg_ ACT	Regulatory complexity; CIA model data only; NOD validation pending (37)

Comparison of dietary indole-3-carbinol, postbiotics, small-molecule AhR agonists, fecal microbiota transplantation, and engineered bacterial therapeutics according to their mechanisms of action, compartments of AhR activation, key advantages, and major limitations.

## 6 Discussion

### 6.1 Reconciling the evidence: the indole paradox as a therapeutic design principle

The evidence synthesized in this review converges on a central mechanistic conclusion: indole is not an inherently tolerogenic molecule. Its immunological valence — whether it restrains or amplifies autoimmunity — is determined by the cellular compartment of action, the innate immune cell identity first encountering the signal, the pre-existing inflammatory milieu of the tissue, and the temporal relationship between metabolite delivery and disease stage. Strategies achieving myeloid-targeted, sustained AhR activation in the lymph node-resident DC and macrophage pool consistently produce tolerogenic outcomes, while those achieving primarily luminal AhR activation at pharmacologically relevant concentrations risk paradoxical Th17 induction (15, 16, 37, 75). The Indole Paradox should therefore be understood not as a toxicological limitation of indole biology, but as a design specification that any viable therapeutic platform must satisfy.

### 6.2 Translational gaps and priority research questions

Several critical gaps remain before any AhR-targeted strategy can advance to T1D clinical trials. First, the context-dependent AhR signaling rules established in CIA and STZ models must be validated in the spontaneous NOD model. Second, the temporal window of AhR-targeted intervention must be established through pharmacokinetic and immunological studies across defined disease stages. Third, the molecular determinants governing tolerogenic versus pro-inflammatory AhR programs in specific APC subsets require definitive characterization through high-dimensional single-cell transcriptomics. Fourth, validated plasma metabolomics biomarkers of the indole/AhR pathway deficit must be developed to enable precision-matched patient stratification.

Sex is an important potential modifier of T1D pathogenesis and responses to immunomodulatory therapy. Female NOD mice develop diabetes more frequently than males, and androgen–microbiome interactions can modify disease susceptibility in this model (18, 19). In humans, this difference is less pronounced and varies with age, with an approximately balanced distribution in childhood and a modest male predominance reported after puberty in some populations (20). Human studies outside the specific setting of T1D have identified sex associated differences in T_reg_ frequency and suppressive function; however, direct evidence that sex modifies therapeutic AhR programming in human T1D is lacking (88). Future preclinical studies should therefore include both sexes and evaluate whether sex influences the efficacy and cellular effects of AhR-targeted interventions.

### 6.3 Positioning AhR-targeted therapies within the T1D therapeutic architecture

Microbiome-targeted AhR-restoration strategies occupy a mechanistically orthogonal and biologically upstream therapeutic niche relative to teplizumab and low-dose IL-2: by restoring the gut-derived indole/AhR/T_reg_ induction axis that primes systemic T_reg_ homeostasis, they address the root cause of the T_reg_ deficit rather than compensating for its consequences (8, 30, 50, 86). The most compelling translational opportunity is combination therapy: microbiome-targeted AhR restoration as an upstream tolerogenic priming intervention, combined downstream with T_reg_-stabilizing therapy such as low-dose IL-2, teplizumab, or adoptive T_reg_ ACT in Stage 1 or Stage 2 T1D patients (89–91).

### 6.4 Regulatory and safety considerations for live biotherapeutic platforms

Clinical development of EBT platforms carries regulatory considerations distinct from conventional small molecules or biologics (92). The FDA regulates LBPs as biological drugs requiring IND submission, phased clinical trials, comprehensive strain characterization, and long-term safety monitoring (92). Synthetic biology provides regulatory-grade containment solutions: auxotrophic mutations, inducible CRISPR-based kill switches validated in clinical-stage *E. coli Nissle* programs, and chromosomal integration of therapeutic transgenes at essential gene loci (80, 93). A specific regulatory consideration applies to *BmΔvjbR::tnaA*: wild-type *B. melitensis* is classified as a BSL-3 pathogen and a CDC/USDA Select Agent, necessitating that the attenuated ΔvjbR strain undergo formal Select Agent exclusion review demonstrating (1): loss of ability to cause brucellosis in immunocompetent hosts (2); *in vivo* clearance kinetics consistent with a non-replicating or rapidly cleared therapeutic; and (3) regulatory-grade manufacturing under containment conditions compliant with 42 CFR Part 73 (94, 95). The documented safety profile of 16MΔvjbR in immunocompromised and pregnant animal models represents a necessary but not sufficient foundation for the IND pathway, and preclinical tolerability studies in non-human primates will be required before this platform can advance to human clinical trials (94, 95).

## 7 Conclusion

The depletion of the indole-AhR-T_reg_ immunoregulatory axis represents a mechanistically upstream and functionally consequential feature of the T1D-associated microbiome dysbiosis. Three principal conclusions emerge from this synthesis. *First*, T1D-associated gut dysbiosis may contribute to impaired immune regulation through altered tryptophan metabolism and reduced availability of selected AhR-active indole metabolites; direct causation of T_reg_ dysfunction in patients remains to be established. *Second*, the immunological outcome of AhR pathway activation is exquisitely context-dependent — governed by ligand identity, delivery route, cellular target, pre-existing inflammatory state, and disease stage — such that dietary or systemic supplemental approaches are pharmacodynamically inadequate and, in the wrong cellular context, actively pro-inflammatory. *Third*, the emerging therapeutic landscape has collectively established proof-of-concept for AhR-targeted tolerance induction and identified the mechanistic conditions under which such induction succeeds or fails. The field now requires systematic validation of leading platforms in the spontaneous NOD model, rigorous mechanistic investigation of the cellular determinants governing AhR-dependent tolerance versus inflammation in APCs and T cells, and clinical biomarker development to stratify T1D patients by indole/AhR pathway deficit for precision-matched therapeutic selection. The indole-AhR-T_reg_ axis offers a mechanistically compelling and therapeutically actionable entry point for precisely this integrated approach to T1D prevention.
