# Supplementary material for: The Indole–AhR–Treg axis as a microbiome-encoded immune checkpoint in type 1 diabetes
Source: Front Immunol. 2026 Oct 30;17:1949305. doi: 10.3389/fimmu.2026.1949305 (PMC13760269; doi:10.3389/fimmu.2026.1949305)
Supplement: Supplementary file 1 [file Supplementaryfile1.docx]

Title: The Indole–AhR–T_reg_ axis as a microbiome-encoded immune checkpoint in type 1 diabetes

*This document contains: Supplementary Note S1 (Figure Construction Methodology); Supplementary Table S1 (Figure 3A Ordinal Scoring Rubric); Supplementary Table S2 (Figure 2C Taxon Evidence Summary); Supplementary Table S3 (Figure 1B Stage-Anchored Derivation); Supplementary Table S4 (Figure 3B Heatmap Cell Assignment Logic).*

**Supplementary Note S1: Figure Construction Methodology, Data Derivation, and Synthesis Rationale**

*Overview*

Several figures in this review are literature-synthesized evidence displays designed to integrate mechanistic and translational findings across heterogeneous experimental systems. In such cases, plotted values represent normalized indices or ordinal evidence rankings rather than raw measurements from a single study. This approach was chosen to avoid false numerical precision when source studies differ substantially in model, platform, taxonomic resolution, and endpoint definition.

Where direct pooled datasets were not available or were not statistically harmonizable across studies, values were normalized or ordinally ranked for visual synthesis in this review and should be interpreted as comparative evidence displays rather than primary quantitative measurements.

*Universal categorization of review figures*

Figures in this review include both evidence-weighted synthesis panels (where plotted values represent normalized indices or ordinal scores synthesized from the cited literature) and conceptual mechanistic schematics; each figure legend specifies its construction approach. The specific categorization and methodology for each figure is documented below.

## Figure 1B — Evidence-weighted synthesis panel (line graph)

*Construction method*: The Foxp3^+^ T_reg_ relative dominance curve and CD4^+^/CD8^+^ T_eff_ curve were constructed by anchoring disease-stage transition timepoints to published NOD mouse immunopathology literature. Stage boundaries (Stages 1–4: weeks 3–5, 8–12, 12–20, 20–30) were taken from Chen et al. (2018), Atkinson et al. (2014), D'Alise et al. (2008), Grebinoski et al. (2024), Ferris et al. (2023), Pearson et al. (2025). T_eff_ dominance trajectories were anchored to CD8^+^ cross-priming data from Ferris et al. (2023) and Pearson et al. (2025). Values represent a synthesized relative dominance index, not absolute cytometric percentages from a single experiment.

Legend note: Y-axis label: "Relative dominance index (%)". Values are illustrative of biological direction and stage-anchored timing; they should not be interpreted as quantitative cytometry data. Error bars and n-values are not applicable to a synthesis figure of this type.

## Figure 2A — Conceptual mechanistic schematic (pathway diagram)

*Construction method:* The three-branch tryptophan catabolism schematic was constructed from KEGG pathway entries for IDO1/IDO2/TDO2 (kynurenine branch), bacterial *TnaA* (indole branch), and TPH1/TPH2 (serotonin branch), cross-referenced with Agus et al. (2018), Koziel & Urbanska (2023), and Lu et al. (2025). Receptor targets and immune outcomes are based on Quintana et al. (2008), Veldhoen et al. (2008), and Haq et al. (2021).

Legend note: This figure is a conceptual schematic. No quantitative data are plotted. Enzyme names, metabolite identities, and receptor targets are directly sourced from the cited literature.

## Figure 2C — Evidence-weighted synthesis panel (grouped bar graph)

*Construction method:* Taxon-level relative abundance shifts were synthesized from the TEDDY cohort (Vatanen et al. 2018), the INNODIA consortium (Vatanen et al. 2024), and supporting studies (Vatanen et al. 2018, Vatanen et al. 2024 (INNODIA)). Because these studies use different 16S variable regions, shotgun vs. amplicon platforms, and different normalization pipelines, raw relative abundance values could not be directly pooled. Instead, a directionally normalized abundance index was computed by: (1) identifying the consistent direction of change (depleted vs. enriched in T1D relative to healthy controls) across ≥2 independent studies; (2) assigning a normalized index value between 10–75 (a.u.) proportional to the magnitude and consistency of reported shifts.

Legend note: Y-axis label: "Directionally normalized abundance index (a.u.)". Values represent comparative evidence displays, not raw 16S read counts from a single dataset. Colors use the Okabe-Ito colorblind-safe palette (blue = Healthy; orange = T1D dysbiotic).

## Figure 3A — Ordinal evidence scoring matrix (grouped bar/lollipop graph)

*Construction method:* Each therapeutic strategy (Dietary I3C, Postbiotics/IPA/IAA, Pharmacological AhR agonists, FMT, EBTs/LBPs) was scored 1–5 across six pharmacological dimensions using a pre-defined ordinal rubric (see Supplementary Table S1). Scores reflect the weight and consistency of available preclinical and clinical evidence for each strategy on each dimension as of the literature cut-off date (March 2026). The EBT translational readiness score was corrected to 2/5 because all published in vivo efficacy data for *BmΔvjbR::tnaA* derive from the DBA/1 collagen-induced arthritis (CIA) model and not the NOD mouse model of T1D.

Legend note: Scores represent ordinal evidence rankings, not pooled quantitative effect sizes. The rubric defining scores 1 and 5 for each dimension is provided in Supplementary Table S1. EBT translational readiness correction is annotated with an asterisk (*) directly on the figure.

## Figure 3B — Conceptual qualitative matrix (heatmap)

Construction method: The context-determinism matrix assigns each cell a qualitative immune valence score (−2 to +2) based on the combination of: ligand identity, delivery compartment, cytokine milieu, T cell activation state, and overall immune outcome. Assignments were made by consensus review of: AhR signaling literature (Quintana 2008, Quintana 2010); dietary I3C paradox data (Kahalehili et al. 2020, Veldhoen et al. 2008); EBT intra-phagocytic delivery data (Das et al. 2022); FMT outcomes (de Groot et al. 2021, Hoyer et al. 2025); and postbiotic IPA/IAA mechanistic studies (Li B et al. 2025, Li Q et al. 2025). Color scale uses the Okabe-Ito blue–yellow diverging palette (blue = tolerogenic; yellow = pro-inflammatory) to ensure colorblind accessibility.

Reviewer caveat / legend note: This figure is conceptual. Colorbar labels are qualitative bins (Strongly tolerogenic, Tolerogenic, Intermediate, Pro-inflammatory, strongly pro-inflammatory) rather than numeric values. Cell assignments should be interpreted as literature-supported directional calls, not quantitative immune measurements. Full cell-level reference assignments are provided in Supplementary Table S4.

**Figure 4: Conceptual mechanistic schematic.** This figure presents a literature-based synthesis of sustained intracellular indole delivery by BmΔvjbR::tnaA and its proposed immunoregulatory effects, based on Das et al. (2022; main manuscript reference 37). The mechanistic and efficacy findings derive from male DBA/1 mice with collagen-induced arthritis. Application to pancreatic lymph nodes and autoimmune diabetes remains hypothetical and requires direct validation in NOD mice.

**Supplementary Table S1: Ordinal Evidence Scoring Rubric for Figure 3A: Comparative Pharmacological Profile of AhR-Targeting Therapeutic Strategies in T1D**

| DIMENSION | SCORE 1 (LOW / ABSENT) | SCORE 5 (HIGH / CONSISTENT) |
| --- | --- | --- |
| Myeloid-targeted delivery | Systemic or luminal delivery only; no evidence of preferential uptake by migratory myeloid cells (monocytes, cDC1, macrophages) at the gut-associated lymphoid tissue interface | Constitutive intraphagocytic delivery to migratory myeloid cells (e.g., CD11b^+^/CD11c^+^ cells) confirmed in vivo; preferential accumulation at draining lymph nodes documented |
| Sustained AhR activation | Single bolus exposure; ligand cleared within 6 hours at target tissue; no evidence of prolonged AhR target gene activation (Cyp1a1, Aldh1a2) | Continuous or prolonged AhR activation confirmed (>24 hours at target site); AhR target gene upregulation sustained across multiple time points in vivo |
| Compartment specificity | Gut-luminal signal only; no systemic AhR activation detected in peripheral lymph nodes (PLN), spleen, or pancreatic draining lymph node | Systemic AhR activation at PLN, spleen, and mesenteric lymph nodes confirmed; evidence of T_reg_ expansion at the islet-proximal immune interface |
| Dosing precision | Highly variable dose delivery; dependent on donor microbiome (FMT), dietary intake, or interindividual metabolic variation; no defined pharmacokinetic/pharmacodynamic (PK/PD) relationship established | Defined, titratable dose with characterized PK/PD relationship; dose-response data available from controlled preclinical or clinical studies |
| T_reg_ induction efficacy | No evidence of Foxp3^+^ T_reg_ expansion in vivo; AhR activation associated with Th17 skewing or neutral immune outcome in disease-relevant model | Robust Foxp3^+^ T_reg_ expansion (≥1.5-fold above control) confirmed in disease-relevant in vivo model; T_reg_ functional suppression assays supporting tolerogenic outcome |
| Translational readiness | Preclinical data only from non-T1D model (e.g., CIA, EAE, STZ); no published NOD mouse, humanized mouse, or human data available in T1D or related autoimmune context | Phase II or Phase III randomized controlled trial (RCT) data available demonstrating safety and/or efficacy in human T1D or a closely related autoimmune disease |

*Each therapeutic strategy was assigned an ordinal score from 1 (low/absent evidence) to 5 (strong/consistent evidence) across six pharmacological dimensions. Intermediate scores (2–4) reflect partial or mixed evidence. Scores represent ordinal rankings synthesized from the literature cited in the main text; they are not pooled quantitative effect sizes.*

*Note: Intermediate scores (2, 3, 4) reflect partial evidence, mixed results across studies, or data from models with limited direct relevance to T1D. The EBT (LBP) translational readiness score of 2/5 reflects efficacy data from the DBA/1 collagen-induced arthritis (CIA) model only (Das et al. 2022); direct NOD mouse and human T1D validation data are required before this score can be increased.*

**Supplementary Table S2: Figure 2C Taxon-Level Evidence Summary: T1D Gut Dysbiosis**

*Supporting literature for each taxon displayed in Figure 2C. Direction indicates consistent finding across ≥2 independent T1D cohort studies. Index value = directionally normalized abundance index (a.u.) assigned for Figure 2C display; not a raw measurement. Studies listed include human T1D pediatric cohorts (TEDDY, INNODIA) and gnotobiotic NOD mouse experiments. H = Healthy control; T1D = Type 1 Diabetes.*

| **TAXON** | **DIRECTION IN T1D** | **INDEX VALUE (H / T1D)** | **SUPPORTING STUDIES** | **BIOLOGICAL RELEVANCE** |
| --- | --- | --- | --- | --- |
| *Faecalibacterium prausnitzii* | Depleted in T1D | 72 (H) / 20 (T1D) | TEDDY (2018), INNODIA (2024) | Anti-inflammatory butyrate producer; TnaA+; Foxp3+ Treg-supportive via SCFA-AhR crosstalk |
| *Akkermansia muciniphila* | Depleted in T1D | 65 (H) / 17 (T1D) | TEDDY (2018), INNODIA (2024) | Mucin-degrading; gut barrier integrity maintenance; inversely correlates with islet autoantibodies |
| *Bifidobacterium spp.* | Depleted in T1D | 60 (H) / 19 (T1D) | TEDDY (2018), INNODIA (2024) | Early-life colonizer; IgA induction; intestinal barrier support; reduced in T1D prior to seroconversion |
| *Prevotella spp.* | Depleted in T1D | 55 (H) / 14 (T1D) | TEDDY (2018), INNODIA (2024) | Associated with anti-inflammatory mucus layer; depleted before first autoantibody in TEDDY cohort |
| *TnaA^+^* anaerobes (indole-producing) | Depleted in T1D | 68 (H) / 14 (T1D) | TEDDY (2018), INNODIA (2024) | TnaA-expressing anaerobes (Clostridiales, Ruminococcaceae) produce AhR ligands IPA and IAA; GADA autoantibodies correlate with reduced Alistipes/tryptophan metabolites |
| *Bacteroides spp.* | Enriched in T1D | 32 (H) / 72 (T1D) | TEDDY (2018), INNODIA (2024) | LPS-producing gram-negative; promotes gut inflammation; elevated in multiple T1D cohorts before clinical onset |
| *Escherichia–Shigella* | Enriched in T1D | 10 (H) / 56 (T1D) | TEDDY (2018), INNODIA (2024) | Gram-negative; endotoxin producer; associated with increased intestinal permeability and systemic immune activation |
| *Proteobacteria* (phylum) | Enriched in T1D | 11 (H) / 49 (T1D) | TEDDY (2018), INNODIA (2024) | Dysbiosis marker phylum; elevated abundance correlates with insulitis severity in NOD mice and human T1D cohorts |

*Abbreviations: TnaA = tryptophanase; IPA = indole-3-propionic acid; IAA = indole-3-acetic acid; GADA = glutamic acid decarboxylase autoantibody; SCFA = short-chain fatty acid; AhR = aryl hydrocarbon receptor; LPS = lipopolysaccharide; H = Healthy control; T1D = Type 1 Diabetes.*

**Supplementary Table S3: Figure 1B NOD Mouse Disease Stage-Anchored Timeline and Data Derivation**

*Stage boundaries, key immunological events, and assigned relative dominance index values used to construct Figure 1B. Values represent a literature-synthesized relative dominance index anchored to published NOD disease-stage timing; they are not raw flow cytometry data from a single cohort. Female NOD mice are used as the primary reference system (60–80% diabetes penetrance by 30 weeks).*

| **STAGE** | **WEEK RANGE** | **KEY IMMUNOLOGICAL EVENTS** | **T_REG_ / T_EFF_ INDEX VALUES** | **T_REG_ TREND** | **T_EFF_ TREND** | **SUPPORTING STUDIES** | **THERAPEUTIC RELEVANCE** |
| --- | --- | --- | --- | --- | --- | --- | --- |
| Stage 1 Peri-insulitis | 3–5 weeks | T_reg_ dominance; peri-islet CD4^+^ T cell infiltration begins; Foxp3^+^ T_regs_ functional but numerically declining; innate immune priming at pancreatic draining LN | 42 / 18 | Declining | Rising | D'Alise et al. (2008); Chen et al. (2018); Atkinson et al. (2014) | Primary AhR intervention window; T_reg_ induction most effective before established insulitis |
| Stage 2 Early insulitis | 8–12 weeks | Progressive islet infiltration; cDC1^-^mediated CD8^+^ cross-priming initiates; T_reg_ frequency declining; T_reg_:T_eff_ crossover ~week 8–9; Th17 cells emerging | 30 / 32 (crossover ~wk 8) | Crossover ~wk 8 | Crossover ~wk 8 | Ferris ST et al. (2023); Pearson et al. (2025); D’Alise et al. (2008) | Secondary AhR intervention window; T_reg_ stabilization still feasible; EBT timing rationale |
| Stage 3 Established insulitis | 12–20 weeks | Established leukocyte infiltration; pathogenic CD8^+^ T cells dominant; IFN-γ/TNF-α inflammatory milieu; β-cell stress responses; islet antigen spreading | 17 / 58 | Declining | Dominant | Kim D et al. (2024); Engin F et al. (2026); Ferraro et al. (2011) | Reduced therapeutic window; AhR agonists may promote Th17 in established cytokine milieu |
| Stage 4 Overt diabetes | 20–30 weeks | β-cell mass <10% of normal; overt hyperglycemia; autoimmune destruction essentially complete; features of chronic T cell activation and dysfunction | 9 / 78 | Minimal | Dominant | Atkinson et al. (2014); Mauvais & van Endert (2025); Engin & Eizirik (2026) | AhR monotherapy insufficient; combination with T_reg_ ACT or anti-CD3 (teplizumab) required |

*Abbreviations: cDC1 = type 1 conventional dendritic cell; LN = lymph node; IFN-γ = interferon-gamma; TNF-α = tumor necrosis factor-alpha; T_eff_ = effector T cell; ACT = adoptive cell therapy; T_reg_ = regulatory T cell; AhR = aryl hydrocarbon receptor.*

**Supplementary Table S4: Figure 3B Heatmap Cell Assignment Logic: Contextual Determinants of AhR-Driven T Cell Fate**

*Qualitative immune valence assignments for each cell of the Figure 3B context-determinism matrix. Valence scale: Strongly tolerogenic (−2), Tolerogenic (−1), Intermediate (0), Pro-inflammatory (+1), Strongly pro-inflammatory (+2). Assignments are based on consensus review of mechanistic and translational literature cited in the main text. References listed are the primary sources supporting each assignment.*

| **^CONTEXT VARIABLE^** | **_STRATEGY_** | **CELL LABEL** | **VALENCE** | **QUALITATIVE BIN** | **ASSIGNMENT RATIONALE** |
| --- | --- | --- | --- | --- | --- |
| Ligand identity | EBTs (LBPs) | IPA/indole sustained | −2 | Strongly tolerogenic | Sustained indole derivatives activate AhR in a TGF-β-dominant context; *BmΔvjbR::tnaA* constitutive production |
| Ligand identity | Pharm. AhR agonists | Synthetic agonist | −1 | Tolerogenic | Endogenous AhR agonists (ITE, FICZ) promote T_reg_; outcome ligand-dependent; TCDD class excluded from therapeutic context |
| Ligand identity | FMT | Restored commensal | 0 | Intermediate | FMT restores diverse indole-producing commensals; ligand mixture produces heterogeneous AhR activation; net outcome intermediate |
| Ligand identity | Postbiotics (IPA/IAA) | IPA/IAA direct | −1 | Tolerogenic | Direct IPA and IAA administration promotes tolerogenic AhR signaling in T cells and DCs |
| Ligand identity | Dietary I3C | I3C/DIM luminal | 0 | Intermediate | I3C/DIM are high-affinity AhR ligands; outcome highly context-dependent; paradox origin |
| Delivery compartment | EBTs (LBPs) | Intraphagocytic LN | −2 | Strongly tolerogenic | EBT intraphagocytic delivery to migratory DCs ensures AhR activation occurs in the draining LN naïve T cell priming environment |
| Delivery compartment | Pharm. AhR agonists | Dose-dep. systemic | −1 | Tolerogenic | Systemic delivery with dose-dependent AhR activation; compartment advantage over dietary but less targeted than EBT |
| Delivery compartment | FMT | Gradual systemic | −1 | Tolerogenic | FMT-mediated microbiota restoration gradually shifts systemic AhR ligand pool; slower kinetics than pharmacological approaches |
| Delivery compartment | Postbiotics (IPA/IAA) | Partial systemic | −1 | Tolerogenic | Oral IPA achieves partial systemic bioavailability; gut barrier-enhancing effect adds complementary mechanism |
| Delivery compartment | Dietary I3C | Luminal only | +2 | Strongly pro-inflammatory | I3C/DIM remain in the gut lumen; systemic bioavailability limited; luminal AhR activation in T1D-relevant IL-6 milieu promotes Th17 |
| Cytokine milieu | EBTs (LBPs) | TGF-β/IL-2 niche | −2 | Strongly tolerogenic | Intraphagocytic delivery ensures AhR activation in TGF-β/IL-2-rich naïve T cell priming niche, strongly biasing toward Foxp3^+^ T_reg_ |
| Cytokine milieu | Pharm. AhR agonists | Context-dep. | 0 | Intermediate | Outcome shifts dramatically with cytokine context; tolerogenic in TGF-β, pro-inflammatory in IL-6; systemic delivery increases context uncertainty |
| Cytokine milieu | FMT | Gradual recovery | −1 | Tolerogenic | FMT gradually restores anti-inflammatory cytokine milieu (SCFA-driven IL-10, TGF-β); not immediately tolerogenic |
| Cytokine milieu | Postbiotics (IPA/IAA) | Partial restore | −1 | Tolerogenic | IPA/IAA partially restore anti-inflammatory milieu; less complete than FMT microbiome normalization |
| Cytokine milieu | Dietary I3C | IL-6/IL-1β dom. | +1 | Pro-inflammatory | In established T1D cytokine milieu (IL-6/IL-1β dominant), luminal I3C-AhR signaling promotes Th17 over T_reg_ |
| T cell activation state | EBTs (LBPs) | Naïve DC priming | −2 | Strongly tolerogenic | Intra-phagocytic LN delivery ensures AhR agonist reaches naïve DCs before T cell activation commitment; optimal for tolerogenic imprinting |
| T cell activation state | Pharm. AhR agonists | Variable | 0 | Intermediate | Systemic AhR agonists encounter both naïve and activated T cells; activated T cells resist tolerogenic reprogramming |
| T cell activation state | FMT | Variable | 0 | Intermediate | FMT-restored commensals interact with both naïve and activated immune compartments; outcome variable |
| T cell activation state | Postbiotics (IPA/IAA) | Mixed | 0 | Intermediate | IPA/IAA metabolites affect both resting and activated T cells; net outcome partially tolerogenic |
| T cell activation state | Dietary I3C | Activated T cells | +1 | Pro-inflammatory | In active insulitis, T cells are pre-activated; luminal I3C-AhR signal insufficient to override activation-driven Th17 commitment |
| Overall immune outcome | EBTs (LBPs) | Tolerogenic / Protected | −2 | Strongly tolerogenic | CIA model: *BmΔvjbR::tnaA* reduced arthritis score, increased Foxp3^+^ T_regs_, decreased Th17; full NOD validation pending |
| Overall immune outcome | Pharm. AhR agonists | ~Tolerogenic / ~Protected | −1 | Tolerogenic | Preclinical evidence for disease protection in EAE, T1D (AGT-5 in STZ); clinical data absent |
| Overall immune outcome | FMT | ~Tolerogenic / ~Protected | −1 | Tolerogenic | Autologous FMT preserved C-peptide better than donor FMT and no AhR mechanism is shown |
| Overall immune outcome | Postbiotics (IPA/IAA) | Partial tolerance | −1 | Tolerogenic | IPA/IAA improve gut barrier, reduce inflammatory cytokines; T1D-specific efficacy data limited to preclinical models |
| Overall immune outcome | Dietary I3C | Th17 / Pro-inflam. | +2 | Strongly pro-inflammatory | NOD mouse data: dietary I3C exacerbated insulitis, expanded Th17, reduced T_reg_; confirmed indole paradox in T1D |

| **Strategy** | **Cells** | **Citations (ref #)** |
| --- | --- | --- |
| EBTs | all five | Das, Gut Microbes, 2022 (37); Quintana, Nature, 2008 (5, ligand and cytokine cells); Takiishi, J Clin Invest, 2012 (81) and Elias-Oliveira, Cell Mol Life Sci, 2025 (82) for the DC-tolerance convergence |
| Pharm. AhR agonists | all five | Yeste, Science Signaling, 2016 (74); Jonic, Frontiers in Immunology, 2024 (75); Quintana, PNAS, 2010 (87); Quintana, Nature, 2008 (5); Veldhoen, Nature, 2008 (29) for the IL-6 case |
| FMT | all five | de Groot, Gut, 2021 (76); Høyer, EClinicalMedicine, 2025 (77) and Diabetologia, 2025 (78) for safety and symptoms only; Tillett, Nature Communications, 2025 (50) for the SCFA/AhR-ligand link |
| Postbiotics | all five | Li Q, Nature Metabolism, 2025 (7); Li B, Gut Microbes, 2025 (58); Wu, Arthritis & Rheumatology, 2026 (57); Chen, Mol Med Rep, 2024 (72); Prasad, Gut, 2026 (73) |
| Dietary I3C | all five | Kahalehili, Frontiers in Immunology, 2020 (15); Veldhoen, Nature, 2008 (29) |

*Row shading: Blue tones = tolerogenic valence (−2 to −1); grey = intermediate (0); yellow tones = pro-inflammatory (+1 to +2). All assignments are literature-supported directional calls; this matrix is conceptual and should not be interpreted as a quantitative meta-analysis. Color bar in Figure 3B uses Okabe-Ito blue, orange and yellow diverging palette for colorblind accessibility.*
